# 

**Published:** 1862-02

**Authors:** 


### HALL'S JOURNAL OF HEALTH.

Vol. 9. FEBRUARY, 1862. No. 2.

### HEALTH TRACTS.

The January and February numbers contain sixty-two one-page Health Tracts. The number of the tract answers to the number of the page. January contains from No. 1 to 31, both included; February has No. 32 to No. 62, both included.

| | Tract |
|---|---|
| Air and Sunshine, | 51 |
| Apples, | 59 |
| Burns and Bites, | 27, 23 |
| Baths and Bathing, | 30 |
| Colds Cured, | 27, 3 |
| Colds Neglected, | 27, 29 |
| Colds Prevented, | 31 |
| Corns and Shoes, | 14 |
| Consumption and Measles, | 11, 16 |
| Coffee Drinking, | 46 |
| Catarrh, | 52 |
| Checking Perspiration, | 58 |
| Costiveness, | 22 |
| Drunkenness and Dyspepsia, | 8 |
| Dyspepsia, | 33 |
| Disease, Causes of, | 4 |
| Disease Avoided, | 28 |
| Drinking, | 35 |
| Diet for Invalids, | 54 |
| Erect Position, | 11 |
| Eating, | 26 |
| Eating Wisely, | 32 |
| Eat, How to, | 34 |
| Eating, When and What, | 40 |
| Eyes, Care of, | 5, 15 |
| Eyesight Failing, | 61 |
| Erysipelas, | 56 |
| Fruits in Summer, | 2 |
| Flannel, Wearing, | 19 |
| Feet, Care of, | 14, 21 |
| Fifteen Follies, | 53 |
| Growing Beautiful, | 15 |
| Hair, the Care of, | 18 |
| Headache, | 41, 62 |
| Health without Medicine, | 20 |
| Healthful Observances, | 38 |
| Health's Three Essentials, | 19, 42 |
| Hydrophobia, | 49 |
| Inconsiderations, | 1 |
| Ice, its Uses, | 9 |
| Music Healthful, | 7 |
| Medicine, Taking, | 60 |
| Nothing but a Cold, | 36 |
| Neuralgia, | 44 |
| Nursing Hints, | 57 |
| Precautions, | 37 |
| Presence of Mind, | 39 |
| Premonitions, | 43 |
| Private Things, | 45 |
| Poisons, | 23, 27, 55 |
| Rheumatism, | 50 |
| Sitting, | 13 |
| Sabbath Physiology, | 17 |
| Sour Stomach, | 24 |
| Sleeping, | 25 |
| Sick-Headache, | 62, 41 |
| Traveling Hints, | 6 |
| The Three Ps, | 47 |
| Winter Rules, | 10 |
| Walking, | 12 |
| Warnings, | 48 |
| Valuable Knowledge, | 27 |

